# Effect of Two Selected Levels of *Padina gymnospora* Biowaste and Enteric Methane Emission, Nutrient Digestibility, and Rumen Metagenome in Growing Sheep

**DOI:** 10.3390/microorganisms13040780

**Published:** 2025-03-28

**Authors:** Archit Mohapatra, Shraddha Trivedi, Chaluvanahalli S. Tejpal, Manojkumar Janardhan Aware, Shalini Vaswani, Vedant Jayeshkumar Prajapati, Atul Purshottam Kolte, Pradeep Kumar Malik, Artabandhu Sahoo, Chandragiri Nagarajarao Ravishankar, Raghavendra Bhatta

**Affiliations:** 1 ICAR-National Institute of Animal Nutrition and Physiology, Bangalore 560030, India; arch10moha@gmail.com (A.M.); shraddha.trivedi_8@yahoo.com (S.T.); vedantprajapati01@gmail.com (V.J.P.); atulkolte@gmail.com (A.P.K.); sahooarta1@gmail.com (A.S.); 2 School of Sciences, JAIN (Deemed-to-Be-University), Bangalore 560027, India; 3 ICAR-Central Institute of Fisheries Technology, Kochi 682029, India; tejpal.arun@gmail.com; 4 BAIF Development Research Foundation, Pune 411058, India; manojkumar.aware@baif.org.in; 5 Uttar Pradesh Pandit Deen Dayal Upadhyaya Pashu Chikitsa Vigyan Vishwavidyalaya Evam Go-Anusandhan Sansthan, Mathura 281001, India; shalini_vet@yahoo.com; 6 ICAR-Central Institute of Fisheries Education, Mumbai 400061, India; cnrs2000@gmail.com; 7 Indian Council of Agricultural Research, New Delhi 110001, India; ragha0209@yahoo.com

**Keywords:** biowaste, brown seaweed, enteric methane, microbiota, *Padina gymnospora*

## Abstract

A study was conducted on growing sheep to investigate the effect of two selected levels of biowaste of *Padina gymnospora* on feed intake, digestibility, daily enteric methane (CH_4_) emission, growth performance, and rumen metagenome. We randomly divided the 18 growing male sheep into three groups of six animals each. The animals were fed on a basal diet comprising finger millet straw (*Eleusine coracana*) and a concentrate mixture in a 35:65 ratio. The sheep in the control group (C) were offered a concentrate mixture without waste, whereas the wheat bran in the concentrate mixture in test group I (A_2_) and test group II (A_5_) was replaced (*w*/*w*) with the biowaste of *Padina gymnospora* at a level of 3.07 and 7.69%, respectively. The biowaste of *Padina gymnospora* at the above levels in concentrate constituted 2 and 5% of the diet. A significant decrease of 28.4% in daily enteric CH_4_ emission (g/d) was reported in the A_5_ group, whereas the difference in daily enteric CH_4_ emission between the C and A_2_ & A_2_ and A_5_ groups did not prove significant. The inclusion of *Padina gymnospora* biowaste did not affect the nutrient intake and digestibility among the groups. The inclusion of *Padina gymnospora* biowaste in the A_5_ group resulted in a significant reduction (*p* = 0.0012) in daily CH_4_ emissions compared with group C; however, no significant differences were observed in daily CH_4_ emissions between groups C–A_2_ (*p* = 0.0793) and A_2_–A_5_ (*p* = 0.3269). Likewise, the adjustment of data to CH_4_ emissions per 100 g of organic matter intake indicated a substantial decrease in the A_5_ group relative to C. The energy loss in CH_4_ as a percentage of GE relative to group C decreased significantly (−23.4%) in the A_5_ group; however, this reduction was not associated with an increase in productivity, as almost similar average daily gain (*p* = 0.827) was observed in the groups. The replacement of wheat bran with the biowaste of *Padina gymnospora* significantly decreased the numbers of total protozoa and *holotrichs* in the A_5_ group. Irrespective of the group, the Bacteroidota was the single largest phylum in the rumen metagenome, representing >60% of the microbiota. However, the abundance of Bacteroidota was similar among the groups. The methanogenic phyla Euryarchaeota was the 5th most abundant; however, it constituted only 3.14% of the metagenome. The abundance of *Desulfovibrio* was significantly higher in the A_5_ group as compared with the control. In conclusion, the significant increase in the abundance of sulfate-reducing bacteria and reduction in protozoal numbers led to a significant reduction in CH_4_ emissions with the incorporation of biowaste of *Padina gymnospora* at a 5% level of the diet.

## 1. Introduction

Though the atmospheric concentration of methane (CH_4_) is far less than that of carbon dioxide, nevertheless, due to its high global warming potential and shorter half-life, CH_4_ is a potent greenhouse gas that has drawn global attention due to its role in climate change. The atmospheric concentration of CH_4_ is about 1934 parts per billion [ppb, World Meteorological Organization, 2024], which is 162% greater than the pre-industrial levels [1]. Irrespective of the sources, annually, 580 Tg (teragram) CH_4_ is produced [2], and at the same time, 571 Tg is removed through various sinks [3]. Thus, every year, there is a net accumulation of around 9 Tg CH_4_ in the atmosphere. Agriculture is one of the largest sectors, contributing a substantial amount of CH_4_ [4]. In the agriculture sector, enteric fermentation, with an annual average emission of 87–97 Tg [5], remains one of the major sources of CH_4_ emissions. Considering the importance of CH_4_ emissions reduction, the global methane pledge was launched at COP26, where many countries endorsed a 30 percent reduction in CH_4_ emissions by 2030. This reduction is not achievable without addressing the enteric CH_4_ emissions. Apart from the global warming perspective, enteric CH_4_ emissions also lead to a sizable loss of energy away from the host animal.

Various CH_4_ mitigation approaches, such as feeding more concentrate [6,7], fat supplementation [8], ionophores [9], tannins [10,11], and saponins [12,13], have been developed and attempted with variable success, largely depending upon the economic status of the livestock farmers, consistency of mitigation impact, adversity on the host animal, rumen microbiota, and feed fermentation. High emissions of CH_4_ from livestock is a global issue, but for economic feasibility and sustainability, they need to be addressed at the local level. Worldwide, there are many research groups working on the development of the most appropriate technologies to tackle enteric CH_4_ emissions. Some of the anti-methanogenic products recently developed are Bovaer, Rumin8, Harit Dhara, and 3-NOP, which are quite promising in reducing emissions. Due to the economic constraint, the adoption rate of the global anti-methanogenic products remains poor in developing countries, where a large scope exists for CH_4_ reduction.

Seaweeds are a rich source of bioactive compounds [14] and are adequately available in coastal countries. There is a huge diversity in the types of seaweeds, and their occurrence is mostly region-specific. *Padina* is a macroalgae belonging to the Dictyotaceae family and is recognized as one of the predominant seaweeds in the Gulf of Mannar region [15]. The Mandapam region is known for its diverse seaweed species and rich marine resources [16]. The Mandapam region has an adequate supply of Padina gymnospora, leading to its applications in the nutraceuticals and hydrocolloids sectors, which results in a significant amount of biowaste from this species. Several reports indicated that the agar extraction process results in the generation of 45–50% of biowaste [17,18]. *Padina gymnospora* is a brown seaweed, and the anti-methanogenic potential of the *Padina gymnospora* or the biowaste obtained from the supercritical fluid extraction was recently established through in vitro studies by our group [19]. Results from the in vitro study indicated a significant decrease in CH_4_ production at the three levels of wheat bran replacement in concentrate with an equal proportion (*w*/*w*) of biowaste of *Padina gymnospora* constituting 2, 5, and 10% of the total diet; however, there was a significant decrease in the dry matter digestibility at the highest level of 10% inclusion [19]. Therefore, the present study chose 2% and 5% incorporation levels for the in vivo evaluation in sheep. The in vitro results are an initial clue and should not be used for recommending the levels of feeding or predicting the emissions/mitigation; therefore, an in vivo study was carried out in growing sheep. We hypothesized that during the processing of *Padina gymnospora* in the nutraceutical industries, various bioactive molecules get concentrated in the biowaste, and the supplementation of biowaste may lead to a reduction in CH_4_ emission through a shift in the rumen microbiota. The present study was conducted with two selected levels of biowaste of *Padina gymnospora* for ascertaining the impact on daily enteric CH_4_ emissions, rumen metagenome, feed digestibility, and growth performance.

## 2. Materials and Methods

### 2.1. Collection of Seaweed and Biowaste

The brown seaweed *Padina gymnospora* was collected by the ICAR-Central Institute of Fisheries Technology (CIFT) from the Mandapam coast of the Indian Ocean, Tamil Nadu, India (9.2770° N, 79.1252° E). After initial rinsing of biomass with fresh water, it was sun-dried in an open, ventilated place. The dried biomass was then transported to the ICAR-CIFT and pulverized, and the selected bioactive compounds, such as phloroglucinol, carotenoid, and fucoxanthin, were extracted using the supercritical fluid extraction technique. The waste generated from the extraction of the above-selected bioactive compounds was briefly rinsed, dried, and ground in a Cyclotec mill (CT293, FOSS, Pune, India) before investigating the impact of graded levels of supplementation of biowaste of *Padina gymnospora* on enteric CH_4_ emissions.

### 2.2. Ethical Approval, Animals, Feeding, and Management

An in vivo experiment was conducted on 18 growing male *Bannur* sheep at the Experimental Livestock Unit of the ICAR-National Institute of Animal Nutrition and Physiology, Bangalore, India (12.97° N and 77.56° E). The Institutional Ethics Committee (IAEC) approved the experiment in sheep vide approval number NIANP/IAEC/1/2024/8. The experiment in growing sheep was conducted in strict compliance with the institutional protocols.

The whole experiment was conducted over a period of 96 days, including 85 days of preliminary feeding and 11 days of CH_4_ measurement and digestibility trials. Eighteen male, growing sheep (mean BW 8.93 ± 0.27 kg) were randomly divided into three groups of six animals each. The animals were fed on a basal diet consisting of finger millet straw (*Eleusine coracana*) and concentrate mixture in the ratio of 35:65. The concentrate mixture was prepared using maize grain (370 g/kg), soybean meal (250 g/kg), groundnut cake (150 g/kg), wheat bran (200 g/kg), mineral mixture (20 g/kg), and salt (10 g/kg). The sheep in control group (C) were offered the concentrate mixture of same composition as described above in this section, whereas the wheat bran in the concentrate mixture for test group I (A_2_) and test group II (A_5_) was replaced (*w*/*w*) with the biowaste of *Padina gymnospora* at the inclusion level of 3.07 and 7.69%, respectively. The inclusion of biowaste of *Padina gymnospora* at the above levels in concentrate replacing wheat bran constituted 2 and 5% of the diet on dry matter basis in A_2_ and A_5_ groups, respectively. The feed was offered to the experimental animals in two equal proportions at 09.00 and 17.00 h.

The animals were housed in a well-ventilated pucca shed in tail-to-tail orientation. The shed has the provision for the feeding and watering of individual animals. Animals had free access to clean drinking water throughout 24 h. Before the commencement of study, all the animals were dewormed with Ivermectin @ 200 mcg per kg of body weight.

### 2.3. Chemical Composition

The dried ground samples of *Padina gymnospora* biowaste, concentrate ingredients, and finger millet straw were analyzed for chemical constituents following the standard procedures. The crude protein (CP) was analyzed by estimating the nitrogen content in feed samples using automatic nitrogen analyzer (VAPODEST 450, Gerhardt, Cäsariusstraße, Germany). To determine the CP, the nitrogen content was multiplied by 6.25. The ash content in the feed samples was estimated by incineration of two-gram sample in a muffle furnace at 550 °C for 4 h, and the organic matter (OM) was calculated by the difference between dried weight of sample taken initially and the ash content. The OM in feed samples was expressed as gram per kg of dry matter. The fiber fractions, viz. neutral detergent fiber (NDF) and acid detergent fiber (ADF), were estimated in accordance with Van Soest et al. [20] using automatic fiber analyzer (Fibretherm FT12, Gerhardt, Cäsariusstraße, Germany).

The gross energy (GE) of the feed samples was determined by a digital bomb calorimeter (RSB 7, Rajdhani Scientific Instrument, New Delhi, India). About 0.5 g ground dried sample was weighed and converted into pellets and then placed into the bomb crucible. The GE was determined by incineration of the sample in a closed oxygen-rich environment, and the rise in temperature due to the combustion of the sample was considered for the calculation of GE. The GE was expressed as megajoule per kilogram of dry matter (MJ/kg).

### 2.4. CH_4_ Measurement

The animals in C, A_2_, and A_5_ groups were fed on the diets outlined above under Section 2.2 for 85 days before the commencement of CH_4_ measurement trial. The mean body weight of sheep among the groups at the commencement of CH_4_ measurement trial was 12.1 ± 0.074 kg (*p* = 0.900). The daily enteric CH_4_ emissions were quantified by employing the sulfur hexafluoride (SF_6_) tracer technique as described by Berndt et al. [21]. The brass permeation tubes (34 mm long, 8.5 mm dia., 30 mm deep, and 4.8 mm blind hole) were initially charged with 812 ± 10.6 milligrams pure SF_6_ gas. A 0.24 mm Teflon septum supported by a 2 μm pore size stainless steel frit was placed in the nut to control the release of gas from the brass tubes served as the source of SF_6_. These permeation tubes were calibrated to achieve constant release over seven weeks at 39 °C while recording the weight of tubes every week. The average release rate of SF_6_ from the permeation tubes at the time of insertion in the rumen was 3.78 ± 0.129 milligrams per day. After the calibration of release rate, the tubes were inserted in the rumen of sheep 10 days prior to the commencement of the CH_4_ measurement trial. The halters were assembled using nylon tube, capillary tube (Supelco, 56712-U, ID 1/16, Darmstadt, Germany), and quick connectors (B-QC4-D-200 and QC4-S-400, Swagelok, Solon, USA) as per Williams et al. [22]. The PVC canister for the gas sampling in the background air was hung daily on the ventilated iron wire mesh fixed in the cement wall in the north direction of the shed. Throughout the CH_4_ measurement trial, the canisters were tied and removed from the individual sheep at a constant fixed time every day. The initial and final pressures of the vacuumized and gassed PVC canisters were measured with a digital pressure meter (Leo 2, Keller, Winterthur, Switzerland). High-purity N_2_ gas was used for the dilution (2.00–3.300 folds) of breath and background samples for the easy successive sub-sampling. The diluted gas samples were injected into the gas chromatograph (GC 2010 plus, Shimadzu, Kyoto, Japan) equipped with a flame ionization detector (FID) and an electron capture detector (ECD) for the estimation of CH_4_ and SF_6_ gasses, respectively. The GC conditions described previously by Malik et al. [23] were upheld for the estimation of both the gasses in the breath samples. The daily enteric CH_4_ emission was calculated using the equation of Moate et al. [24]. Due to the complexity of SF_6_ assembly attributed to the halters, PVC canisters, dilution, pressure, and gas chromatography, there is substantial inconsistency in the day-to-day CH_4_ measurement [25]. That is why the CH_4_ observations are not recorded by days but rather counted by successful collections. A minimum of seven successful collections of the breath samples from individual sheep were ensured during the trial. The CH_4_ emission was expressed as g/d, g/100 g DM, g/100 g digestible DM, g/100 g OM and g/100 g digestible OM.

### 2.5. Nutrient Intake and Digestibility

Concurrent with the CH_4_ measurement, the digestibility trial was conducted in sheep. The daily feed allowance for the individual sheep was weighed and offered in the manger. Similarly, the feed refusals and fecal output from the feeding of previous day were weighed for the individual animal, and the representative samples were collected for the nutrient analysis. The feed, refusals, and feces samples were dried at 80 °C for 24 h. Thereafter, the samples were ground for the estimation of chemical composition as outlined under Section 2.3. The nutrient intake (g/d) was calculated by subtracting the nutrient in feed refusals from the nutrient in feed offered, whereas the apparent nutrient digestibility (%) was determined by considering the nutrient intake and the nutrient voided through feces. The sample collection, representative sample for DM estimation, N content in fresh and dry dung, and preservation of fresh dung in sulfuric acid were performed as described previously [26]. The feed, refusals, and feces samples for GE estimation were dried at 60 °C.

### 2.6. Rumen Fermentation and Protozoa

At the end of experiment, the rumen digesta was collected through stomach connected to a vacuum pump (Mityvac 8000, Lincoln Industrial, St. Louis, MO, USA). Prior to the actual collection, about 30 mL rumen digesta was collected and discarded to avoid saliva contamination. Thereafter, about 45 mL rumen digesta was collected from the individual sheep at 4 h post-feeding. The rumen digesta through stomach tube was collected in an airtight collection vessel [27] and divided into three subsets of 15 mL each for the genomic DNA (1st set), ammonia, and VFA estimation (2nd set), and protozoal enumeration (3rd set). The 1st and 2nd subsets after collection were immediately placed in the ice box, whereas the 3rd subset was transported to the normal environmental temperature.

The 2nd subset of rumen digesta from the individual sheep was transferred to the individual tubes and centrifuged at 13,400 rpm for 15 min at 4 °C. The supernatant obtained was equally divided into halves, where first half of the supernatant was used for VFA estimation, and the remaining half was used for the estimation of ammonia-N. For VFA estimation, one part of 25% metaphosphoric acid was added to the four parts of supernatant and preserved at 4 °C till analysis. The VFAs were estimated according to Filípek and Dvorak [28] using a gas chromatograph (7890B GC, Agilent, Baden-Württemberg, Germany) following the conditions previously described by Malik et al. [29,30].

For the estimation of ammonia-N, a few drops of saturated HgCl_2_ were added before preserving the supernatant at 4 °C until analysis. After thawing, the ammonia-N in the supernatant was determined using the method of Conway [31] as described previously [23]. Third subset of rumen digesta was used for the enumeration of protozoa. In brief, 1 mL of rumen fluid was mixed with 1 mL of 37% formaldehyde and kept at room temperature overnight. The protozoa were identified based on the morphology/presence of the cilia, categorized according to Hungate [32], and enumerated under a phase-contrast microscope (Ci-S, Nikon, Tokyo, Japan) as per Kamra and Agarwal [33].

### 2.7. Growth

To investigate the impact of the supplementation of selected levels of *Padina gymnospora* biowaste replacing equal parts of wheat bran in the concentrate mixture on the productive performance, the body weight of individual sheep was recorded regularly at the two-week interval during the entire experimental period. The DM intake (g/d) was monitored for the individual animal and modified weekly according to the change in body weight of sheep. The average daily gain (ADG) was calculated considering the change in body weight and duration of experimental period. The ADG was expressed as grams per day.

### 2.8. Microbial Diversity

First subset of the rumen digesta was used for DNA isolation. Approximately 1.5 mL of the digesta containing both solid and liquid fractions from individual sheep was transferred to a 2 mL Eppendorf tube. The DNA from the rumen digesta was isolated according to the procedure of Yu and Morrison [34] employing repeat bead beating plus column (RBB+C) method. The rumen digesta samples were centrifuged at 12,000× *g* for 15 min, and the supernatant was carefully removed and discarded. Thereafter, 1 mL lysis buffer was added to dissolve the pellet. The dissolved content was transferred to a pre-sterilized screw-cap tube (BioSpec Products, Bartlesville, OK, USA) containing 0.5 g zirconia beads of 0.1 mm size (Cat. 11079101z, BioSpec, Bartlesville, USA). The samples were homogenized in a mini bead beater (Mini Bead Beater 24, BioSpec, Bartlesville, USA) at the maximum speed for 3 min. Subsequently, the content was incubated at 70 °C for 15 min, followed by centrifugation at 12,000× *g*. The supernatant was collected in a 2 mL Eppendorf tube, and about 300 μL lysis buffer was added to the screw-cap tube containing residue after the removal of supernatant. The bead-beating process, as stated above, was again performed, and the supernatant was removed and pooled with the previously removed supernatant. For the precipitation of proteins and polysaccharides, the supernatant was treated with 260 μL of 10 M ammonium acetate, followed by incubation on the ice for 5 min. Thereafter, the centrifugation was carried out at 12,000× *g*, 4 °C for 10 min. The supernatant was removed, and an equal volume of isopropanol was added and mixed by gentle inversion. The DNA was collected by centrifugation at 4 °C for 10 min at 12,000× *g*, and then the pellet was washed with 70% ethanol. The DNA pellet was dissolved in 100 μL Tris-EDTA buffer, followed by the addition of 2 μL DNase-free RNase (10 mg/mL, Qiagen GmbH, Hilden, Germany) to remove the RNA contamination. After this step, the QIAamp DNA mini kit (Cat. 51306, Qiagen GmbH, Hilden, Germany) was used following the manufacturer’s instructions. The quality of metagenomic DNA was checked with 0.8% agarose gel electrophoresis and quantified by Qubit 4.0 (Thermofisher, Carlsbad, CA, USA).

The shotgun sequencing of metagenomic DNA was performed on NovaSeq 6000 (Illumina Inc., San Diego, CA, USA) at Eurofins Genomics, Bangalore, India. The metagenomic libraries were prepared using NEBNext^®^ Ultra^TM^ II FS DNA Library Prep Kit (New England Biolabs, Ipswich, MA, USA). The known quantity of metagenomic DNA (100–500 ng) was fragmented to achieve a size of 350 base pairs. The fragmentation process was carried out using NEBNext Ultra II FS Reaction Buffer and Ultra II FS Enzyme Mix in a PCR thermal cycler. The fragmented DNA was ligated with the NEBNext Adaptor for Illumina through the process of combining 35 μL of fragmented DNA with the NEBNext Ultra II Ligation Master Mix. The mixture was then incubated at 20 °C for 15 min, followed by ligation with adaptors. Thereafter, the PCR amplification was carried out using index primers (i5 and i7) under the following PCR conditions: initial denaturation at 98 °C, 30 s, denaturation at 98 °C, 10 s, annealing at 65 °C, 75 s, and final extension at 65 °C, 5 min. The PCR-enriched libraries were evaluated on Agilent 4150 Tape Station and then sequenced on NovaSeq 6000 to generate paired-end reads of 150 base pairs length.

The metagenomic raw reads were screened for quality and adapter contamination using FastQC v0.11.9. The adapters, low-quality bases (Q < 30), and reads shorter than <100 bp were removed using Trimmomatic v0.39 [35] with the following parameters: ILLUMINACLIP:TruSeq3-PE-2.fa:2:30:10SLIDINGWINDOW:15:30 MINLEN:100 TRAIL- ING:30 AVGQUAL:30. The clean reads obtained after quality filtration in Trimmomatic were screened in BowTie2 v2.5.0 [36] and preconfigured for the removal of contamination with human, mouse, and PhiX reads. The host contamination (sheep) was removed in BowTie2 v2.5.0 using the custom target database Oar_v4.0 (RefSeq assembly accession: GCF_000298735.2). The clean reads obtained after the removal of contamination from human, mouse, PhiX, and sheep reads were uploaded in BV-BRC v 3.30.19 [37] and taxonomically classified following the K-mer approach in Kraken2 [38]. The resultant output was parsed into taxonomic levels in Pavian v1.2.0 [39]. The data were normalized using total sum scaling (TSS) and analyzed in MicrobiomeAnalyst v2.2 [40]. The annotated data at different taxonomic ranks were analyzed in MicrobiomeAnalyst v2.2 [40] by using the default count filter of four reads. The feature read counts were clustered [41] and presented based on taxonomic ranks, i.e., at the phylum, order, and genus levels. The metagenome data at different taxonomic ranks among the groups were compared using the Kruskal–Wallis test, and the mean values with significance were ascertained using the Dunn post-hoc test in the rstatix package in R v4.3.1. The alpha diversity was assessed using the Shannon index, whereas the beta diversity was measured through the Bray–Curtis dissimilarity index at the genus level in MicrobiomeAnalyst v2.2.

### 2.9. Statistical Analysis

The data were checked for the Gaussian distribution using Kolmogorov–Smirnov test at the 5% significance level in GraphPad Prism version 10.2.3 (GraphPad Software, San Diego, CA, USA). Data on CH_4_ emission, nutrient intake, digestibility, growth, and fermentation parameters were by one-way ANOVA using following mathematical model:

$$Y_{ij}=µ+\tau_{i}+\epsilon_{ij}$$

where *Y_ij_* represents the *j*th observation (*j* = 1, 2, …… 10) on the *i*th treatment (*i* = 1, 2, 3). µ was the common effect of the experiment, $\tau_{i}$ represents the *i*th treatment effect, and *ϵ_ij_* represents the random error due to the *j*th observation of the *i*th treatment. 

## 3. Results

### 3.1. Chemical Composition

The chemical composition of ragi straw and three concentrate mixtures fed to the sheep in C, A_2_, and A_5_ groups is presented in Table 1. All three concentrate mixtures were isocaloric and contained ~16.9 MJ per kilogram of dry matter. Similarly, the crude protein (CP) was also in the narrow range of 21.5–22.2 percent in the concentrate. The neutral detergent fiber (NDF) and acid detergent fiber (ADF) content of the concentrate mixture in groups A_2_ and A_5_ was higher than the NDF and ADF in the C group. Similarly, the ash content in the concentrate mixture of the corresponding groups was considerably higher than in group C, and therefore, the organic matter (OM) content in the concentrate mixture of A_2_ and A_5_ was lower.

### 3.2. CH_4_ Emissions

**Figure 1:**
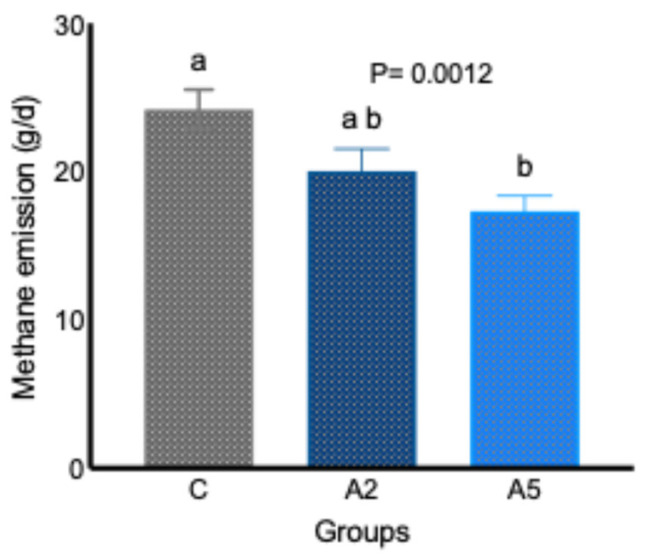
Effect of two levels of biowaste of *Padina gymnospora* supplementation on daily enteric CH_4_ emission in growing sheep. C—control without biowaste supplementation; A_2_—test treatment I with 2% biowaste of *Padina gymnospora*; A_5_—test treatment II with the biowaste of *Padina gymnospora* inclusion at 5% level; g/d—gram per day. Superscripts a and b represent the significant mean values within a row.

Data from the in vivo study indicated that the inclusion of the biowaste of *Padina gymnospora* in the concentrate replacing equal parts of wheat bran decreased (*p* = 0.0012) the daily enteric CH_4_ emission (g/d, Figure 1). A significant decrease (*p* = 0.0012) of 28.4 percent in daily enteric CH_4_ emission (g/d) was reported in the A_5_ group, whereas the difference in daily enteric CH_4_ emission between the C and A_2_ (*p* = 0.0793) and A_2_ and A_5_ (*p* = 0.3269) groups did not prove significant. The adjustment of CH_4_ data to per 100 g intake of DM revealed a significantly lower (*p* = 0.0019) emission in test groups A_2_ and A_5_ (Table 2), whereas adjustment of data to per 100 g of OM intake demonstrated a significant (*p* = 0.0028) reduction in A_5_ group. The CH_4_ emission per 100 g dig. DM intake was also significantly lower (*p* = 0.0001) in both A_2_ and A_5_ groups; however, there was a significant difference (*p* = 0.015) in the CH_4_ emission between the C and A_5_ groups when the data w corrected to per 100 g of dig. OM intake (Table 2). There was no difference in CH_4_ emission per 100 g of OM intake between the C and A_2_, A_2_, and A_5_ groups. Data on CH_4_ emission indicated a consistent reduction in A_5_ as compared with group C in different expression units (daily CH_4_ g/d, CH_4_ g/100 g DM, and CH_4_ g/100 g OM).

### 3.3. Nutrient Intake and Digestibility

The nutrient intake (g/d and g/kg BW) was similar among the groups and not affected with the inclusion of biowaste of *Padina gymnospora* at 2 (A_2_) and 5 (A_5_) percent levels (Table 2) of the diet replacing wheat bran in the concentrate in equal proportions. Similarly, the digestibility of nutrients was also not affected by the incorporation of the test source at the selected levels. Similarly, there was no difference in nutrient digestibility among the groups.

**Figure 2:**
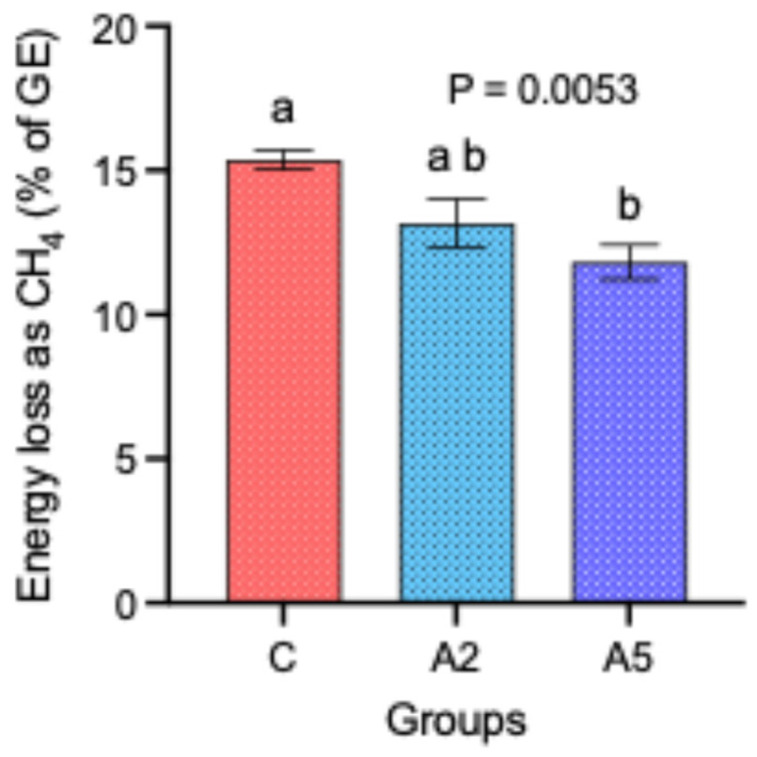
Effect of two levels of biowaste of *Padina gymnospora* supplementation on the energy loss (% of GE) in the form of CH_4_ in growing sheep. C—control without biowaste supplementation; A_2_—test treatment I with 2% biowaste of *Padina gymnospora*; A_5_—test treatment II with the biowaste of *Padina gymnospora* inclusion at 5% level; GE—gross energy. Superscripts a and b on the top of bar represent the significant mean values.

Though there was no difference in the DM intake and the calorific value of the concentrate offered to the sheep in the C, A_2_, and A_5_ groups, nevertheless, the energy loss as CH_4_ percent of the gross energy (GE) was significantly decreased by 23.4 percent in the A_5_ group as compared with C (Figure 2). There was no significant difference in the energy loss between the other groups, i.e., C–A_2_ and A_2_–A_5_.

### 3.4. Rumen Fermentation and Protozoa

The incorporation of biowaste of *Padina gymnospora* replacing wheat bran in concentrate and constituting 2 and 5 percent of the diet did not affect the ruminal ammonia (*p* = 0.099) and TVFA (*p* = 0.080) production as compared with control (Table 3). Similarly, there was no shift in the production of major volatile fatty acids such as acetate, propionate, and butyrate. The iso-butyrate concentration (mmol/L) in the test groups A_2_ and A_5_ was significantly lower (*p* = 0.002) than that in group C. Similarly, a decreasing (*p* = 0.018) concentration of valerate was recorded in the test groups. The replacement of wheat bran with the biowaste of *Padina gymnospora* significantly decreased the numbers of total protozoa (*p* = 0.001) and *Holotrichs* (*p* < 0.0001) in the A_5_ group, whereas there was no difference between the C and A_2_.

### 3.5. Growth

**Figure 3:**
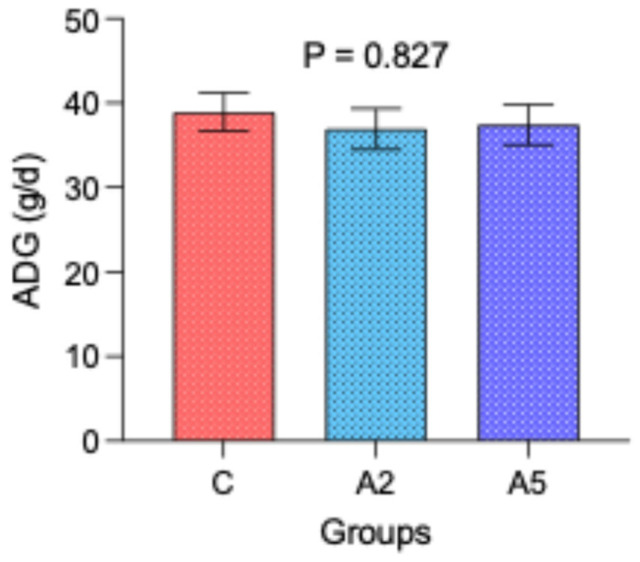
Effect of biowaste of *Padina gymnospora* supplementation on average daily gain (ADG). C—control without biowaste supplementation; A_2_—test treatment I with 2% biowaste of *Padina gymnospora*; A_5_—test treatment II with the biowaste of *Padina gymnospora* inclusion at 5% level.

The similar initial (mean 8.87 kg, *p* = 0.980) and final body weight (mean 12.1 kg, *p* = 0.900) among the groups resulted in the comparable average daily gain (*p* = 0.827, Figure 3) over 12 weeks of the experiment. These results indicated that the significant reduction in enteric CH_4_ emission and lower gross energy loss as CH_4_ in the A_5_ group as compared with C could not be translated into production.

### 3.6. Microbial Diversity

**Figure 4:**
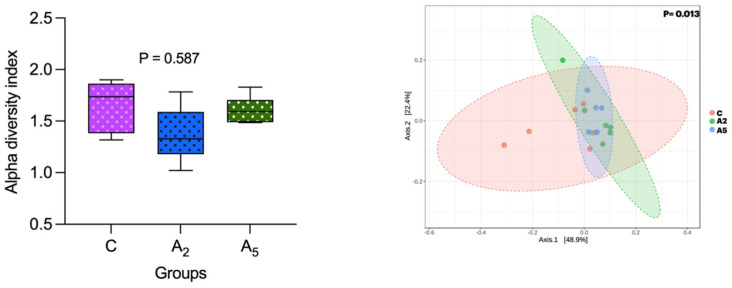
Effect of biowaste of *Padina gymnospora* supplementation on alpha and beta diversity. C—control without biowaste supplementation; A_2_—test treatment I with 2% biowaste of *Padina gymnospora*; A_5_—test treatment II with the biowaste of *Padina gymnospora* inclusion at 5% level.

Alpha diversity ascertained through the Shannon index did not reveal a significant difference (*p* = 0.587) between the samples within the group (Figure 4). However, there was a significant difference (*p* = 0.013) in the beta diversity, revealing the diversity difference between the groups. There was a significant difference (*p* = 0.003) in the microbial diversity between C and A5 groups, whereas the beta diversity between C and A_2_ (*p* = 0.084) and A_2_ and A_5_ (*p* = 0.214) groups was non-significant.

In this study, a total of 1281 million reads with an average of 427 million per group and 71 million per sample were generated. After trimmoatic filtration, 4.84 percent of reads per group were dropped due to the low quality or short length. Further, 03.4 percent of the reads were removed due to a host contamination. The rumen microbes affiliated to 22 phyla, 63 orders, and 320 genera were identified by the Kraken2 database.

**Figure 5:**
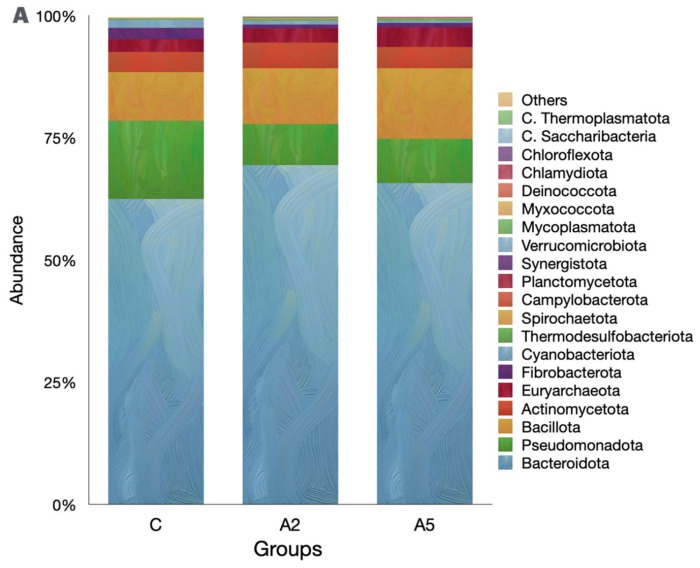

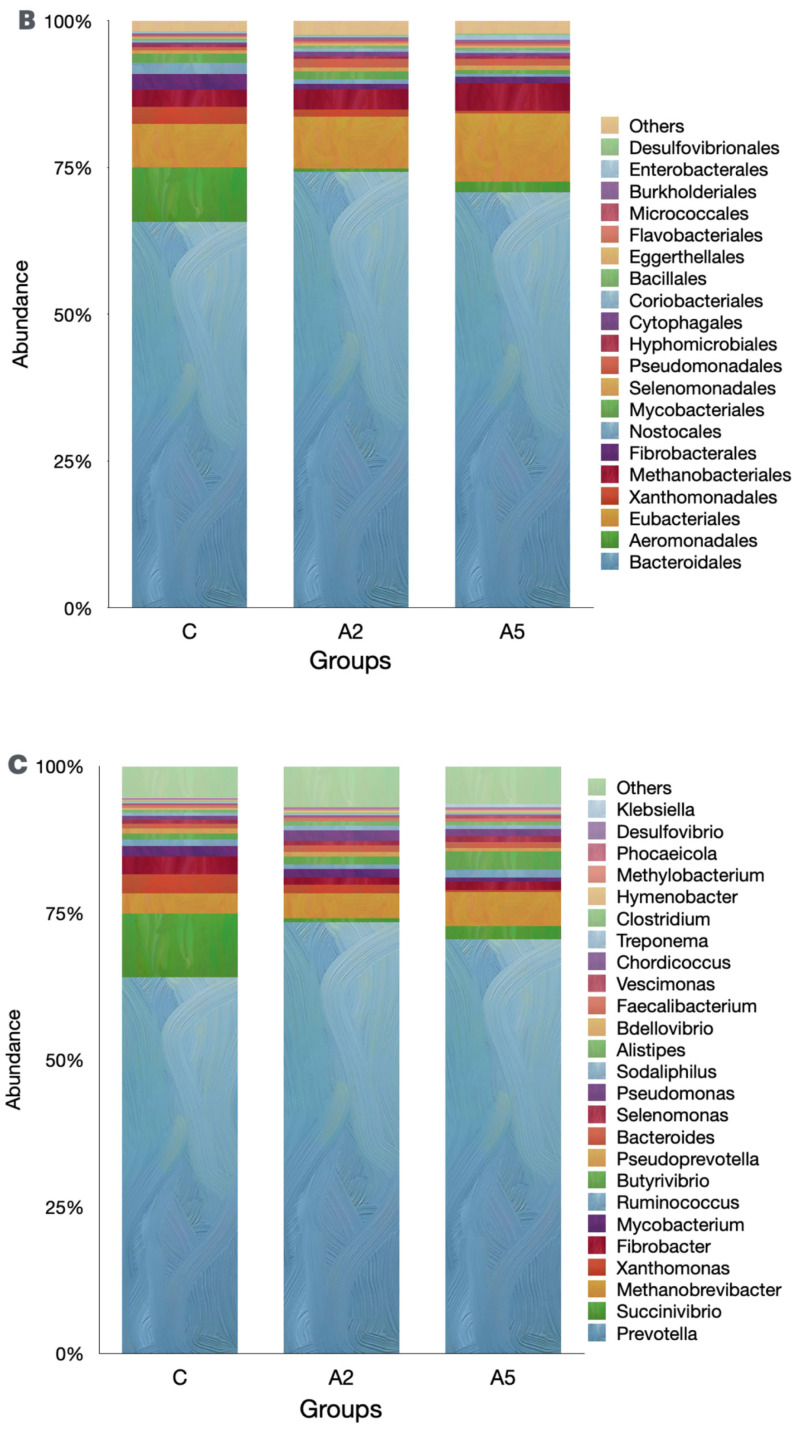
Effect of biowaste of *Padina gymnospora* supplementation on rumen metagenome at three different taxonomic ranks: (**A**) at the phylum level, (**B**) at the order level, and (**C**) at the genus level. The top 20 phyla and orders are visualized in panels (**A**) and (**B**), respectively, whereas the top 25 genera are visualized in panel (**C**). C—control without biowaste supplementation; A_2_—test treatment I with 2% biowaste of *Padina gymnospora*; A_5_—test treatment II with the biowaste of *Padina gymnospora* inclusion at 5% level.

Irrespective of the group, the Bacteroidota was the single largest phylum in the rumen metagenome (Figure 5A, Suppl 1), representing >60 percent of the microbiota. However, the abundance of Bacteroidota was similar (*p* = 0.309) among the groups. Pseudomonadota was next the abundant phylum in the metagenome, and their abundances were adversely affected (*p* = 0.045) by the inclusion of the biowaste of *Padina gymnospora* in the A_2_ and A_5_ groups. On the contrary, there was an increase (*p* = 0.166) in the abundance of the third largest phylum, Bacillota; however, the increased abundance was not proved significant as compared with group C. The methanogen phylum Euryarchaeaota was the 5th most abundant; however, they constituted only 3.14 percent of the metagenome. The abundance of Euryarchaeaota was not different (*p* = 0.484) among the groups. The decreasing abundance of Cyanobacteria (*p* = 0.0102) was reported in the biowaste groups A_2_ and A_5_ as compared with group C. On the contrary, the abundance of Thermodesulfobacteriota was increased (*p* = 0.0179) in group A_5_ as compared with C. At the order level, Bacteroidales were the most abundant; however, there was no difference (*p* = 0.166) in the abundance among the groups (Figure 5B). The orders with the significant decrease in the abundances were Xanthomonadales (*p* = 0.0233), Nostocales (*p* = 0.0189), Mycobacteriales (*p* = 0.025), and Desulfuromonadales (*p* = 0.0073). On the contrary, the abundances of microbes affiliated with Micrococcales (*p* = 0.0464), Desulfovibrionales (*p* = 0.038), and Synechococcales (*p* = 0.0464) were increased in the test groups.

At the genus level, *Prevotella* was the most abundant; however, there was no difference (*p* = 0.203) in the abundance among the groups (Figure 5C). The abundances of *Xanthomonas*, *Mycobacterium*, *Nostoc*, and *Calothrix* genera were decreased. On the contrary, the abundance of *Desulfovibrio* was increased in the A_5_ group as compared with the control (*p* = 0.0342, Suppl 1). The relative abundance of *Desulfovibrio* in A_2_ was higher than in group C, but it was not significant. *Methanobrevibacter* was the 3rd largest genus in the rumen metagenome and was the most dominant among the archaeal genera (Suppl 1). However, the abundance of rumen methanogens, including *Methanobrevibacter*, was not affected by the biowaste of *Padina gymnospora* in the present study.

## 4. Discussion

The abatement of enteric CH_4_ emissions from livestock is the top priority of animal scientists. The search for the suitable and most appropriate feeding technology is perpetual for widening the basket of anti-methanogenic technologies. The paper reported the impact of the biowaste of *Padina gymnospora* on enteric CH_4_ emissions in sheep.

The overall depression in feed digestibility is one of the possible mechanisms for the reduction in enteric CH_4_ emissions. CH_4_ is an output of carbohydrate fermentation, especially of complex carbohydrates in fibrous diets in the rumen [42,43]. Any depression in the carbohydrate fermentation, partially in fiber degradation, leads to less CH_4_ output [8,44,45]. However, the similar intake and nutrient digestibility in growing sheep among the groups ruled out any implication for enteric CH4 emissions. The shift in the VFA proportion is another mechanism by which the total CH_4_ output is altered. The conversion of acetate to propionate led to less CH_4_ output; however, no such conversion of acetate to propionate was apparent in our study, implying that the shift in the VFA towards propionate production was not the reason behind the reduction in enteric CH_4_ emissions. Conversion of acetate to propionate is regulated by the partial H_2_ pressure [46]. The presence of sulfur might be one of the reasons for the non-conversion of acetate to propionate, as Laanbroek et al. [47] also did not report the conversion of acetate to propionate in the presence of sulfate. The sulfur content (2.71–3.52 mg/kg) in the biowaste of *Padina gymnospora* in the present study was probably adequate to check the conversion of acetate to propionate, as evidenced by the similar concentration of both acetate and propionate across the groups. The similar VFA concentration and other fermentation characteristics indicated that the compositional shift in rumen fermentation was not accountable for the reduction in enteric CH_4_ emission due to the feeding of the biowaste of *Padina gymnospora*.

The direct inhibition of methanogens is well known for the significant reduction in the rumen methanogenesis [48,49,50]. A wide diversity of methanogens affiliated with three distinct groups—hydrogenotrophic, methylotrophic, and aceticlastic—are reported in the rumen of various livestock species [51,52,53,54,55]. However, in a previous study, the KEGG analysis revealed the absence of the aceticlastic methanogenesis pathway in Indian sheep fed on an almost similar diet [56]. The dominance of hydrogenotrophic *Methanobrevibacter* is well aligned with the previous report in sheep [56]. The metagenome data did not imply any compositional difference in the archaeal community among the groups; therefore, the direct inhibition of the methanogens by the biowaste of *Padina gymnospora* is excluded for the reduction in CH_4_ emissions.

One of the plausible explanations for the significant reduction in enteric methanogenesis is the higher abundance of sulfate-reducing bacteria in the rumen of the A_5_ group, as evidenced by the metagenome data. Sulfate-reducing bacteria (SRB) are known to outcompete methanogens for the utilization of H_2_, a common substrate for both the SRB and methanogens. The methanogens utilize H_2_ for the reduction in CO_2_ to CH_4_, whereas the SRB uses H_2_ for the reduction in sulfate to H_2_S. Smith et al. [57], from a study, concluded that with the abundant sulfate and H_2_, the SRB has 106 times less half-saturation constant than the methanogens, and that is why they are capable of outcompeting methanogens. The abundance of *Desulfovibrio* was significantly higher in _the A5_ group as compared with the control. In consonance with [58,59,60], others also reported an increase in the abundance of *Desulfovibrio* with the increased sulfate content in the rumen. In the present study, the abundance of *Desulfovibrio* was 2.11 and 2.87 times increased in groups A_2_ and A_5_, respectively, as compared with the control. However, the relative abundance of *Desulfovibrio* was still within the reported range of <1% of the microbiota [59]. Although the current study did not quantify the H_2_S concentration, earlier reports [61,62] confirm a concentration of 0.52 to 1.07 g/m^3^ in the sheep rumen fed on dried distillers’ grains. The excessive concentration of H_2_S is reported to cause respiratory, enteric, and encephalic ailments [63,64]. A higher concentration of H_2_S in the rumen beyond 2000 mg/L [64] may lead to the development of polioencephalomalacia and the death of the animal [65]. However, an old report revealed no toxic and side effects of H_2_S on animal health at a concentration below 471.2 mg/L [66]. These findings warrant further investigation for quantifying the concentration of H_2_S in rumen fed on the biowaste of *Padina gymnospora* or other seaweeds in the long term.

Apart from the direct inhibition of methanogens, obstructing the H_2_ supply to methanogens could be another equally important but indirect mechanism to achieve a reduction in CH_4_ emissions [10]. Protozoa are recto and endo symbiotically associated with the methanogens and transfer H_2_ for the reduction in CO_2_ to CH_4_. Thus, the protozoa are the major microbes that supply one of the most desired substrates to the methanogens. Reduction in the numbers of protozoa by any means is partially checking the supply of obligatory substrate (H_2_). Protozoa are considered unwarranted members of the rumen microbiota [67]. They contribute to the fiber digestion in the rumen [68]. However, some of the reports concluded either an increase [69,70] or no impact on fiber digestion [71,72] in the partial absence of rumen protozoa. Nevertheless, their role in the interspecies H_2_ transfer makes them an important member of the microbial community, which helps in maintaining the syntrophy in the rumen ecosystem. A decrease in the number of protozoa in both the A_2_ and A_5_ groups indicated the adverse impact of the biowaste of *Padina gymnospora* on the rumen protozoa, which in turn affected the CH_4_ emissions in the test groups.

Although there was a significant reduction of 28 percent in enteric CH_4_ emissions in the A_5_ group and a decreasing trend in CH_4_ emissions as compared with control in the A_2_ group, nevertheless, the energy saved from the reduced emissions was not translated into the production as there was no difference in the ADG among the groups. These findings demonstrated that the mitigation of emissions does not transpose into production improvement. In a recent review, Morgavi et al. [73] opined that under the moderate inhibition of ~25%, the improvement in net energy availability for production is difficult considering energy flows in the system. The spared H_2_ from the CH_4_ mitigation in this study was probably used by the SRB, as evidenced by their significantly higher abundance rather than the shift towards propionate production, which is used as an energy source for productive functions.

A comparison of in vivo results with the previous in vitro study [19] with similar inclusion levels of 2 and 5% of biowaste *Padina gymnospora* revealed that the extent of CH_4_ mitigation in growing sheep was 50–75% of the reduction achieved in vitro. The deviation in CH_4_ mitigation extent between the in vivo and in vitro studies can be attributed to the type of systems. The rumen is inherently dynamic in nature, and the fermented digesta is instantly removed, whereas the in vitro system is static. In the dynamic rumen system (in vivo), the metabolites are either directly absorbed through the rumen epithelium, taken up by the microbiota, or passed through the lower tract. On the contrary, there is no provision for the epithelial absorption of the metabolites in the in vitro system or passing through the lower tract; therefore, they accumulate in the system itself. Thus, the in vitro results should be used as a clue rather than the extrapolation and making recommendations.

*Asparagopsis taxiformis* is one of the largely studied seaweeds reported to achieve a greater reduction in CH_4_ emission [74]. Some of the recent studies in cattle [74,75] reported a ~30–70% reduction in CH_4_ emissions at a supplementation level of 0.25–0.50% of the diet. However, Li et al. [76] and Romero et al. [77] reported a reduction of 80% and 34% in CH_4_ emission in sheep and goats at a supplementation level of 3% of OM and 0.5% of the diet, respectively. These findings established that *Asparagopsis taxiformis* is comparatively more effective than the biowaste of *Padina gymnospora*, even at the lower level of supplementation. This deviation in the CH_4_ mitigation extent could be due to the high bromoform content in *Asparagopsis taxiformis*; however, we did not come across any study reporting the bromoform in *Padina gymnospora*.

## 5. Conclusions

From the results, it can be inferred that the replacement (*w*/*w*) of wheat bran with the biowaste of *Padina gymnospora* seaweed in the concentrate equivalent to 5 percent of the diet can decrease the daily enteric CH_4_ emissions by ~28 percent in the growing sheep. A significant increase in the abundance of sulfate-reducing bacteria and a reduction in the protozoal numbers led to a significant reduction in CH_4_ emissions with the incorporation of biowaste of *Padina gymnospora* at a 5 percent level of the diet. However, the output product of sulfate reduction (H_2_S) needs to be quantified in future studies to support the findings of this study. At this level, the biowaste of the above brown seaweed does not impact the nutrient intake, digestibility, and rumen fermentation adversely. However, the supplementation of biowaste at 5 percent could not exhibit any improvement in the growth rate in sheep. The feeding of biowaste shall be adopted in the coastal areas where the brown seaweed is adequately available to mitigate the enteric CH4 from livestock with a minimum input cost. However, further long-term studies are warranted in large ruminants to ascertain the impact of feeding biowaste on milk production and composition and confirm if any of the bioactive compounds are excreted in the milk.

## Figures and Tables

**Table 1:** Chemical composition (g/kg DM) of experimental diets.

Attributes	Concentrate			Ragi Straw	Biowaste *
	C	A_2_	A_5_		
OM	937	921	906	927	786
CP	215	219	222	35.2	139
NDF	378	386	391	733	690
ADF	98.7	105	124	514	433
TA	63.1	78.5	94.4	73.4	214
GE (MJ/kg)	16.99	16.96	16.95	16.48	19.0

g/kg DM—gram per kilogram dry matter; OM—organic matter; CP—crude protein; NDF—neutral detergent fiber; ADF—acid detergent fiber; TA—total ash; C—control treatment without biowaste supplementation; A_2_—test treatment I supplemented with 2% biowaste of *Padina gymnospora*; A_5_—test treatment II supplemented with the biowaste of *Padina gymnospora* inclusion at the 5% level. Sulfur (S) content in biowaste was 2.71–3.52 mg/kg. * refers to the biowaste of *Padina gymnospora*.

**Table 2:** Effect of *Padina gymnospora* biowaste on enteric CH_4_ emission, intake and digestibility, and fermentation.

Attributes	C	A_2_	A_5_	SEM	*p*
CH_4_ emissions					
g/100 DM	4.96 ^a^	4.11 ^b^	3.53 ^b^	0.415	0.0019
g/100 g OM	5.28 ^b^	4.43 ^ab^	3.82 ^a^	0.423	0.0028
g/100 g dig DM	6.67 ^a^	5.54 ^b^	4.88 ^b^	0.522	0.0001
g/100 g dig. OM	7.12 ^b^	6.14 ^ab^	5.60 ^a^	0.444	0.0232
Intake (g/d)					
DM	493	496	493	1.201	0.994
OM	463	459	455	2.309	0.825
CP	81.3	82.2	78.4	1.146	0.577
NDF	239	253	251	4.371	0.648
ADF	111	132	123	6.082	0.07
Digestibility (%)					
DM	73.6	73.1	72.2	0.409	0.067
OM	73.3	69.7	69.8	1.183	0.124
CP	76.9	75.9	75.3	0.466	0.826
NDF	62.8	57.2	62.4	1.803	0.154
ADF	49.5	46.7	50.1	1.047	0.710

C—control without biowaste supplementation; A_2_—test treatment I with 2% biowaste of *Padina gymnospora*; A_5_—test treatment II with the biowaste of *Padina gymnospora* inclusion at 5% level. DM—dry matter, OM—organic matter, CP—crude protein, NDF—neutral detergent fiber, ADF—acid detergent fiber, g/100 g dig—grams per 100 g digestible, SEM—standard error of mean, *p*—measure of significance at 5% probability. Superscripts a and b represent the significant mean values within a row.

**Table 3:** Effect of *Padina gymnospora* biowaste supplementation on rumen fermentation and protozoa.

Attributes	C	A_2_	A_5_	SEM	*p*
Ammonia-N (mg/dL)	14.5	11.8	10.5	1.178	0.0993
TVFA (mmol/L)	81.2	83.9	81.0	0.929	0.9227
Acetate (mmol/L)	54.2	57.9	56.5	1.078	0.8034
Propionate (mmol/L)	16.5	18.3	17.5	0.520	0.6624
Butyrate (mmol/L)	8.21	6.28	6.10	0.677	0.1861
Iso-butyrate (mmol/L)	0.638 ^b^	0.177 ^a^	0.021 ^a^	0.185	0.0002
Valerate (mmol/L)	0.806 ^b^	0.535 ^ab^	0.265 ^a^	0.156	0.0182
Isovalerate (mmol/L)	0.910	0.662	0.703	0.076	0.3604
Total protozoa (cells × 10^7^/mL)	22.3 ^b^	18.8 ^b^	11.4 ^a^	3.219	0.0014
Holotrichs (cells × 10^6^/mL)	1.32 ^b^	1.27 ^b^	0.58 ^a^	0.239	<0.0001

C—control without biowaste supplementation; A_2_—test treatment I with 2% biowaste of *Padina gymnospora*; A_5_—test treatment II with the biowaste of *Padina gymnospora* inclusion at 5% level. Ammonia-N—ammonia nitrogen, TVFA—total volatile fatty acids, mmol/L—millimoles per liter, mL—milliliter, SEM—standard error of mean, *p*—measure of significance at 5% probability. Superscripts a and b represent the significant mean values within a row.

## Data Availability

The microbiota datasets presented in this study can be found in online repositories with the accession numbers PRJNA1208085. The metagenome data with the accession number(s) are available in the repository/repositories and can be found at https://www.ncbi.nlm.nih.gov/sra/PRJNA1208085 (accessed on 25 February 2025).

## References

[1] NOAA Increase in Atmospheric Methane Set Another Record During 2021. https://www.noaa.gov/news-release/increase-in-atmospheric-methane-set-another-record-during-2021.

[2] IEA (2024). Global Methane Tracker 2024.

[3] Jackson R.B., Saunois M., Bousquet P., Canadell J.G., Poulter B., Stavert A.R., Bergamaschi P., Niwa Y., Segers A., Tsuruta A. (2020). Increasing Anthropogenic Methane Emissions Arise Equally from Agricultural and Fossil Fuel Sources. Environ. Res. Lett..

[4] Saunois M., Stavert A.R., Poulter B., Bousquet P., Canadell J.G., Jackson R.B., Raymond P.A., Dlugokencky E.J., Houweling S., Patra P.K. (2020). The Global Methane Budget 2000–2017. Earth Syst. Sci. Data.

[5] Chang J., Peng S., Ciais P., Saunois M., Dangal S.R.S., Herrero M., Havlík P., Tian H., Bousquet P. (2019). Revisiting Enteric Methane Emissions from Domestic Ruminants and Their Δ13CCH4 Source Signature. Nat. Commun..

[6] van Lingen H.J., Niu M., Kebreab E., Valadares Filho S.C., Rooke J.A., Duthie C.A., Schwarm A., Kreuzer M., Hynd P.I., Caetano M. (2019). Prediction of Enteric Methane Production, Yield and Intensity of Beef Cattle Using an Intercontinental Database. Agric. Ecosyst. Environ..

[7] Arndt C., Hristov A.N., Price W.J., McClelland S.C., Pelaez A.M., Cueva S.F., Oh J., Dijkstra J., Bannink A., Bayat A.R. (2022). Full Adoption of the Most Effective Strategies to Mitigate Methane Emissions by Ruminants Can Help Meet the 1.5 °C Target by 2030 but Not 2050. Proc. Natl. Acad. Sci. USA.

[8] Beauchemin K.A., Kreuzer M., O’Mara F., McAllister T.A. (2008). Nutritional Management for Enteric Methane Abatement: A Review. Aust. J. Exp. Agric..

[9] Duffield T.F., Rabiee A.R., Lean I.J. (2008). A Meta-Analysis of the Impact of Monensin in Lactating Dairy Cattle. Part 2. Production Effects. J. Dairy Sci..

[10] Bhatta R., Uyeno Y., Tajima K., Takenaka A., Yabumoto Y., Nonaka I., Enishi O., Kurihara M. (2009). Difference in the Nature of Tannins on in Vitro Ruminal Methane and Volatile Fatty Acid Production and on Methanogenic Archaea and Protozoal Populations. J. Dairy Sci..

[11] Malik P.K., Uyeno Y., Kolte A.P., Kumar R., Trivedi S., Bhatta R. (2019). Screening of Phyto-Sources from Foothill of Himalayan Mountain for Livestock Methane Reduction. SN Appl. Sci..

[12] Jayanegara A., Wina E., Takahashi J. (2014). Meta-Analysis on Methane Mitigating Properties of Saponin-Rich Sources in the Rumen: Influence of Addition Levels and Plant Sources. Asian-Australas. J. Anim. Sci..

[13] Malik P.K., Singhal K.K. (2008). Saponin Content of Lucerne Fodder and Its Effect on Rumen Fermentation and Microbial Population in Crossbred Bulls. Indian J. Anim. Sci..

[14] Lomartire S., Marques J.C., Gonçalves A.M.M. (2022). An Overview of the Alternative Use of Seaweeds to Produce Safe and Sustainable Bio-Packaging. Appl. Sci..

[15] Patel N., Banafarr P., Ramachandran P., Ghosh A., Johnson B., Dharani G. (2024). Strategy for the Development of Seaweed Value Chain: Fostering Diversified Livelihoods.

[16] Ravi P., Subramanian G. (2017). Biochemical Studies on Marine Algal Species of Padina (Phaeophyceae) from Mandapam Coastline, Tamil Nadu, India. World J. Pharm. Res..

[17] Tůma S., Izaguirre J.K., Bondar M., Marques M.M., Fernandes P., da Fonseca M.M.R., Cesário M.T. (2020). Upgrading End-of-Line Residues of the Red Seaweed Gelidium Sesquipedale to Polyhydroxyalkanoates Using Halomonas Boliviensis. Biotechnol. Rep..

[18] Cebrián-Lloret V., Metz M., Martínez-Abad A., Knutsen S.H., Ballance S., López-Rubio A., Martínez-Sanz M. (2022). Valorization of Alginate-Extracted Seaweed Biomass for the Development of Cellulose-Based Packaging Films. Algal Res..

[19] Mohapatra A., Trivedi S., Kolte A.P., Tejpal C.S., Elavarasan K., Vaswani S., Malik P.K., Ravishankar C.N., Bhatta R. (2024). Effect of Padina Gymnospora Biowaste Inclusion on in Vitro Methane Production, Feed Fermentation, and Microbial Diversity. Front. Microbiol..

[20] Van Soest P.J., Robertson J.B., Lewis B.A. (1991). Methods for Dietary Fiber, Neutral Detergent Fiber, and Nonstarch Polysaccharides in Relation to Animal Nutrition. J. Dairy Sci..

[21] Berndt A., Boland T.M., Deighton M.H., Gere J.I., Grainger C., Hegarty R.S., Iwaasa A.D., Koolaard J.P., Lassey K.R., Luo D., Lambert M. (2014). Guidelines for Use of Sulphur Hexafluoride (SF6) Tracer Technique to Measure Enteric Methane Emissions from Ruminants.

[22] Williams S.R.O., Moate P.J., Deighton M.H., Lambert M.G. (2014). Sampling Background Air. Guidelines for Use of Sulphur Hexaflouride (SF6) Tracer Technique to Measure Enteric Methane Emissions from Ruminants.

[23] Malik P.K., Trivedi S., Mohapatra A., Kolte A.P., Sejian V., Bhatta R., Rahman H. (2021). Comparison of Enteric Methane Yield and Diversity of Ruminal Methanogens in Cattle and Buffaloes Fed on the Same Diet. PLoS ONE.

[24] Moate P.J., Williams S.R.O., Deighton M.H., Pinares-Patiño C., Lassey K.R., Lambert M.G. (2014). Estimating Methane Emission Rates and Methane Yield Using the SF6 Technique. Guidelines for Use of Sulphur Hexaflouride (SF6) Tracer Technique to Measure Enteric Methane Emissions from Ruminants.

[25] Pinares-Patiño C.S., Clark H. (2008). Reliability of the Sulfur Hexafluoride Tracer Technique for Methane Emission Measurement from Individual Animals: An Overview. Aust. J. Exp. Agric..

[26] Malik P.K., Trivedi S., Kolte A.P., Mohapatra A., Biswas S., Bhattar A.V.K., Bhatta R., Rahman H. (2023). Comparative Rumen Metagenome and CAZyme Profiles in Cattle and Buffaloes: Implications for Methane Yield and Rumen Fermentation on a Common Diet. Microorganisms.

[27] Malik P.K., Soren N.M., Thulasi A., Prasad C.S. (2015). Simple Method for Rumen Content Collection from 2 Days Old Lambs. Indian Vet. J..

[28] Filípek J., Dvořák R. (2009). Determination of the Volatile Fatty Acid Content in the Rumen Liquid: Comparison of Gas Chromatography and Capillary Isotachophoresis. Acta Vet. Brno.

[29] Malik P.K., Trivedi S., Kolte A.P., Mohapatra A., Biswas S., Bhattar A.V.K., Bhatta R., Rahman H. (2023). Comparative Analysis of Rumen Metagenome, Metatranscriptome, Fermentation and Methane Yield in Cattle and Buffaloes Fed on the Same Diet. Front. Microbiol..

[30] Malik P.K., Trivedi S., Mohapatra A., Kolte A.P., Mech A., Victor T., Ahasic E., Bhatta R. (2024). Oat Brewery Waste Decreased Methane Production and Alters Rumen Fermentation, Microbiota Composition, and CAZymes Profiles. Microorganisms.

[31] Conway E.J. (1957). Microdiffusion Analysis and Volumetric Error.

[32] Hungate R.E. (1966). The Rumen and Its Microbes.

[33] Kamra D.N., Agarwal N. (2003). Techniques in Rumen Microbiology.

[34] Yu Z., Morrison M. (2004). Improved Extraction of PCR-Quality Community DNA from Digesta and Fecal Samples. Biotechniques.

[35] Bolger A.M., Lohse M., Usadel B. (2014). Trimmomatic: A Flexible Trimmer for Illumina Sequence Data. Bioinformatics.

[36] Langmead B., Salzberg S.L. (2012). Fast Gapped-Read Alignment with Bowtie 2. Nat. Methods.

[37] Olson R.D., Assaf R., Brettin T., Conrad N., Cucinell C., Davis J.J., Dempsey D.M., Dickerman A., Dietrich E.M., Kenyon R.W. (2023). Introducing the Bacterial and Viral Bioinformatics Resource Center (BV-BRC): A Resource Combining PATRIC, IRD and ViPR. Nucleic Acids Res..

[38] Wood D.E., Lu J., Langmead B. (2019). Improved Metagenomic Analysis with Kraken 2. Genome Biol..

[39] Breitwieser F.P., Salzberg S.L. (2020). Pavian: Interactive Analysis of Metagenomics Data for Microbiome Studies and Pathogen Identification. Bioinformatics.

[40] Chong J., Liu P., Zhou G., Xia J. (2020). Using MicrobiomeAnalyst for Comprehensive Statistical, Functional, and Meta-Analysis of Microbiome Data. Nat. Protoc..

[41] Paulson J.N., Colin Stine O., Bravo H.C., Pop M. (2013). Differential Abundance Analysis for Microbial Marker-Gene Surveys. Nat. Methods.

[42] Doreau M., Benhissi H., Thior Y.E., Bois B., Leydet C., Genestoux L., Lecomte P., Morgavi D.P., Ickowicz A. (2016). Methanogenic Potential of Forages Consumed throughout the Year by Cattle in a Sahelian Pastoral Area. Anim. Prod. Sci..

[43] Jiyana S.T., Ratsaka M.M., Leeuw K.J., Mbatha K.R. (2022). Impacts of Graded Dietary Fiber Levels on Feed Efficiency and Carbon Footprint of Two Beef Breeds. Livest. Sci..

[44] McSweeney C.S., Palmer B., McNeill D.M., Krause D.O. (2001). Microbial Interactions with Tannins: Nutritional Consequences for Ruminants. Anim. Feed. Sci. Technol..

[45] Haque M.N., Hansen H.H., Storm I.M.L.D., Madsen J. (2017). Comparative Methane Estimation from Cattle Based on Total CO_2_ Production Using Different Techniques. Anim. Nutr..

[46] Van Lingen H.J., Plugge C.M., Fadel J.G., Kebreab E., Bannink A., Dijkstra J. (2016). Thermodynamic Driving Force of Hydrogen on Rumen Microbial Metabolism: A Theoretical Investigation. PLoS ONE.

[47] Laanbroek H.J., Abee T., Voogd I.L. (1982). Alcohol Conversion by Desulfobulbus Propionicus Lindhorst in the Presence and Absence of Sulfate and Hydrogen. Arch. Microbiol..

[48] Denman S.E., Tomkins N.W., McSweeney C.S. (2007). Quantitation and Diversity Analysis of Ruminal Methanogenic Populations in Response to the Antimethanogenic Compound Bromochloromethane. FEMS Microbiol. Ecol..

[49] Mcsweeney C., Kang S., Gagen E., Davis C., Morrison M., Denman S. (2009). Recent Developments in Nucleic Acid Based Techniques for Use in Rumen Manipulation. Rev. Bras. De Zootec..

[50] Altermann E., Schofield L.R., Ronimus R.S., Beatty A.K., Reilly K. (2018). Inhibition of Rumen Methanogens by a Novel Archaeal Lytic Enzyme Displayed on Tailored Bionanoparticles. Front. Microbiol..

[51] Wright A.D.G., Williams A.J., Winder B., Christophersen C.T., Rodgers S.L., Smith K.D. (2004). Molecular Diversity of Rumen Methanogens from Sheep in Western Australia. Appl. Environ. Microbiol..

[52] Wright A., Toovey A., Pimm C. (2006). Molecular Identification of Methanogenic Archaea from Sheep in Queensland, Australia Reveal More Uncultured Novel Archaea. Anaerobe.

[53] Janssen P.H., Kirs M. (2008). Structure of the Archaeal Community of the Rumen. Appl. Environ. Microbiol..

[54] Snelling T.J., Genç B., McKain N., Watson M., Waters S.M., Creevey C.J., Wallace R.J. (2014). Diversity and Community Composition of Methanogenic Archaea in the Rumen of Scottish Upland Sheep Assessed by Different Methods. PLoS ONE.

[55] Pitta D.W., Indugu N., Melgar A., Hristov A., Challa K., Vecchiarelli B., Hennessy M., Narayan K., Duval S., Kindermann M. (2022). The Effect of 3-Nitrooxypropanol, a Potent Methane Inhibitor, on Ruminal Microbial Gene Expression Profiles in Dairy Cows. Microbiome.

[56] Malik P.K., Trivedi S., Kolte A.P., Mohapatra A., Bhatta R., Rahman H. (2022). Effect of an Anti-Methanogenic Supplement on Enteric Methane Emission, Fermentation, and Whole Rumen Metagenome in Sheep. Front. Microbiol..

[57] Smith N.W., Shorten P.R., Altermann E., Roy N.C., McNabb W.C. (2020). Competition for Hydrogen Prevents Coexistence of Human Gastrointestinal Hydrogenotrophs in Continuous Culture. Front. Microbiol..

[58] Zhao Y., Xie B., Gao J., Zhao G. (2020). Dietary Supplementation with Sodium Sulfate Improves Rumen Fermentation, Fiber Digestibility, and the Plasma Metabolome through Modulation of Rumen Bacterial Communities in Steers. Appl. Environ. Microbiol..

[59] Wu H., Li Y., Meng Q., Zhou Z. (2021). Effect of High Sulfur Diet on Rumen Fermentation, Microflora, and Epithelial Barrier Function in Steers. Animals.

[60] Antal V., Andriana C., Nicolae C., Kévin G., Christine D. (2023). Effects of Sulfur Sources on Ruminal S Bioavailability, Fermentation Activity and Microbial Populations Measured In Vitro. Bull. Univ. Agric. Sci. Vet. Med. Cluj-Napoca. Anim. Sci. Biotechnol..

[61] Neville B.W., Schauer C.S., Karges K., Gibson M.L., Thompson M.M., Kirschten L.A., Dyer N.W., Berg P.T., Lardy G.P. (2010). Effect of Thiamine Concentration on Animal Health, Feedlot Performance, Carcass Characteristics, and Ruminal Hydrogen Sulfide Concentrations in Lambs Fed Diets Based on 60% Distillers Dried Grains plus Solubles. J. Anim. Sci..

[62] Neville B.W., Lardy G.P., Karges K.K., Schauer C.S. (2011). Sulfur Intake, Excretion, and Ruminal Hydrogen Sulfide Concentrations in Lambs Fed Increasing Concentrations of Distillers Dried Grains with Solubles. Goat Res. J. Sheep Goat Res. J..

[63] Gooneratne S.R., Christensent D.A. (1989). Review of Copper Deficiency and Metabolism in Ruminants. Can. J. Anim. Sci..

[64] Gould D.H., Cummings B.A., Hamar D.W. (1997). In Vivo Indicators of Pathologic Ruminal Sulfide Production in Steers with Diet-Induced Polioencephalomalacia. J. Vet. Diagn. Investig..

[65] Delfiol D.J.Z., Cunha P.H.J.D., Borges A.S. (2011). Determination of Ruminal Hydrogen Sulfide in Sheep. Vet. Zootec..

[66] Lewis D. (1954). The Reduction of Sulphate in the Rumen of the Sheep. Biochem. J..

[67] Morgavi D.P., Martin C., Jouany J.P., Ranilla M.J. (2012). Rumen Protozoa and Methanogenesis: Not a Simple Cause-Effect Relationship. Br. J. Nutr..

[68] Ushida K., Kayouli C., De Smet S., Jouany J.P. (1990). Effect of Defaunation on Protein and Fibre Digestion in Sheep Fed on Ammonia-Treated Straw-Based Diets with or without Maize. Br. J. Nutr..

[69] Soetanto H., Gordon G.L., Hume I.D., Leng R.A. The Role of Protozoa and Fungi in Fibre Digestion in the Rumen of Sheep. Proceedings of the 3rd AAAP Animal Science Congress, Efficient Animal Production for Asian Welfare.

[70] Romulo B.H., Bird S.H., Leng R.A. (1986). The Effects of Defaunation on Digestibility and Rumen Fungi Counts in Sheep Fed High-Fibre Diets. Proc. Aust. Soc. Anim. Prod..

[71] Jouany J.P. (1996). Effect of Rumen Protozoa on Nitrogen Utilization by Ruminants. J. Nutr..

[72] Han C.Y., Lu D.X., Hu M., Tan Z.L. (1999). Influence of Controlling Protozoa on the Degradation and Utilization of Dietary Fibre and Protein in the Rumen and Nitrogenous Flow Entering the Duodenum of Sheep. Asian-Aust. J. Anim. Sci..

[73] Morgavi D.P., Cantalapiedra-Hijar G., Eugène M., Martin C., Noziere P., Popova M., Ortigues-Marty I., Muñoz-Tamayo R., Ungerfeld E.M. (2023). Review: Reducing Enteric Methane Emissions Improves Energy Metabolism in Livestock: Is the Tenet Right?. Animal.

[74] Roque B.M., Salwen J.K., Kinley R., Kebreab E. (2019). Inclusion of Asparagopsis Armata in Lactating Dairy Cows’ Diet Reduces Enteric Methane Emission by over 50 Percent. J. Clean. Prod..

[75] Angellotti M., Lindberg M., Ramin M., Krizsan S.J., Danielsson R. (2025). Asparagopsis Taxiformis Supplementation to Mitigate Enteric Methane Emissions in Dairy Cows—Effects on Performance and Metabolism. J. Dairy Sci..

[76] Li X., Norman H.C., Kinley R.D., Laurence M., Wilmot M., Bender H., De Nys R., Tomkins N. (2018). Asparagopsis Taxiformis Decreases Enteric Methane Production from Sheep. Anim. Prod. Sci..

[77] Romero P., Ungerfeld E.M., Popova M., Morgavi D.P., Yáñez-Ruiz D.R., Belanche A. (2024). Exploring the Combination of Asparagopsis Taxiformis and Phloroglucinol to Decrease Rumen Methanogenesis and Redirect Hydrogen Production in Goats. Anim. Feed Sci. Technol..

